# José Saramago’s “Blindness” and a Vision for Mental Healthcare: perspectives in the fields of Literature, Architecture, Philosophy, Politics and Economics

**DOI:** 10.1192/j.eurpsy.2023.379

**Published:** 2023-07-19

**Authors:** B. Jorge, J. Carvalho

**Affiliations:** Hospital de Braga, Braga, Portugal

## Abstract

**Introduction:**

The Portuguese writer and Nobel prize winner José Saramago, is well-known for his sharp depiction and reflection of human condition. The recent events of the COVID pandemic juxtapose with his novel “Blindness” (1995) – original title: “Ensaio sobre a Cegueira”–, where the expression of fear of dehumanization in a globalized world where any contemporary society may lead to the obligation to follow what power structures define and establish. In his epidemic of blindness, an abandoned psychiatric asylum was chosen by the author as a quarantine ward and the centre of the plot. A question imposes: why an asylum? What is the focus of such a place in a cultural postmodernism message?

**Objectives:**

An historic background revision is proposed, glancing at the evolution of the architectural concept of asylum evolved until modern times, while setting a reflection towards today’s mental health services and European models.

**Methods:**

A narrative review was performed, gathering points of view in the fields of Literature, Architecture, Philosophy, Politics and Economics.

**Results:**

Bertolt Brecht claims that “all art is political and the question is simply whether art attacks existing structures of power or refuses such attacks and thereby contributes to the continuation of those structures”. Regarding evolution of Asylum Architecture, and the principles which ought to control Modern Construction, In “Blindness”, the thematic of space appears above all through the reference of Marc Augé’s Non-Place. Initially extended to the city, gives way to the funneling of the space that leads to the “asylum” - a space that centralizes all the action. Through Saramago’s description, the floor plans were designed by Portuguese architect José Cardoso. As the first waves of blind people are imprisoned, it is characterized as a heterotopia, the embodiment of Foucaudian panopticism, as it is constituted as a prison whose role is to isolate, even if this attitude is motivated by despair of the government. A mental healthcare system assumes a multidisciplinary approach to psychiatric disorders. Evidence points to a balance between community-based and modern hospital-based care, with frontline services based in the community and hospitals playing a more specialized role. For most European countries, mental healthcare is financed in the same way as other healthcare services, using either national, regional or local budgets and four ways to purchase mental health services are looked at in depth. Therefore, general decisions about such financing may not be in line with mental health policy-maker or planner.

**Conclusions:**

Where culture meets anthropology, social policies, legal boundaries and ethic reflections, a time for a joint dialogue arises. How to surpass the differences and heterogeneity between countries? Is there a place for a common system in mental health care?

**Disclosure of Interest:**

None Declared

